# Paired Associative Stimulation as a Tool to Assess Plasticity Enhancers in Chronic Stroke

**DOI:** 10.3389/fnins.2019.00792

**Published:** 2019-08-02

**Authors:** Joshua Silverstein, Mar Cortes, Katherine Zoe Tsagaris, Alejandra Climent, Linda M. Gerber, Clara Oromendia, Pasquale Fonzetti, Rajiv R. Ratan, Tomoko Kitago, Marco Iacoboni, Allan Wu, Bruce Dobkin, Dylan J. Edwards

**Affiliations:** ^1^Human Motor Recovery Laboratory, Burke Neurological Institute, White Plains, NY, United States; ^2^Department of Rehabilitation and Human Performance, Icahn School of Medicine at Mount Sinai, New York, NY, United States; ^3^Sant Joan de Deu Hospital, Department of Neurology, University of Barcelona, Barcelona, Spain; ^4^Department of Healthcare Policy and Research, Weill Cornell Medical College, New York, NY, United States; ^5^Department of Neurology, Weill Cornell Medical College, New York, NY, United States; ^6^Memory Evaluation and Treatment Service, Burke Rehabilitation Hospital, White Plains, NY, United States; ^7^Burke Neurological Institute, White Plains, NY, United States; ^8^Feil Family Brain and Mind Research Institute, Weill Cornell Medical College, New York, NY, United States; ^9^Department of Psychiatry and Biobehavioral Sciences, UCLA Semel Institute for Neuroscience and Human Behavior, Los Angeles, CA, United States; ^10^Ahmanson-Lovelace Brain Mapping Center, University of California, Los Angeles, Los Angeles, CA, United States; ^11^Department of Neurology, University of California, Los Angeles, Los Angeles, CA, United States; ^12^Department of Neurology, Geffen School of Medicine, Reed Neurologic Research Center, University of California, Los Angeles, Los Angeles, CA, United States; ^13^Moss Rehabilitation Research Institute, Elkins Park, PA, United States; ^14^School of Medical and Health Sciences, Edith Cowan University, Perth, WA, Australia

**Keywords:** paired associative stimulation, stroke, memantine, neurorehabilitation, TMS

## Abstract

**Background and Purpose:**

The potential for adaptive plasticity in the post-stroke brain is difficult to estimate, as is the demonstration of central nervous system (CNS) target engagement of drugs that show promise in facilitating stroke recovery. We set out to determine if paired associative stimulation (PAS) can be used (a) as an assay of CNS plasticity in patients with chronic stroke, and (b) to demonstrate CNS engagement by memantine, a drug which has potential plasticity-modulating effects for use in motor recovery following stroke.

**Methods:**

We examined the effect of PAS in fourteen participants with chronic hemiparetic stroke at five time-points in a within-subjects repeated measures design study: baseline off-drug, and following a week of orally administered memantine at doses of 5, 10, 15, and 20 mg, comprising a total of seventy sessions. Each week, MEP amplitude pre and post-PAS was assessed in the contralesional hemisphere as a marker of enhanced or diminished plasticity. Strength and dexterity were recorded each week to monitor motor-specific clinical status across the study period.

**Results:**

We found that MEP amplitude was significantly larger after PAS in baseline sessions off-drug, and responsiveness to PAS in these sessions was associated with increased clinical severity. There was no observed increase in MEP amplitude after PAS with memantine at any dose. Motor threshold (MT), strength, and dexterity remained unchanged during the study.

**Conclusion:**

Paired associative stimulation successfully induced corticospinal excitability enhancement in chronic stroke subjects at the group level. However, this response did not occur in all participants, and was associated with increased clinical severity. This could be an important way to stratify patients for future PAS-drug studies. PAS was suppressed by memantine at all doses, regardless of responsiveness to PAS off-drug, indicating CNS engagement.

## Introduction

The capacity of the brain to make structural, physiological, and genetic adaptations following stroke, otherwise known as plasticity, is likely to be critical for improving sensorimotor impairments and functional activities. Promotion of adaptive plasticity in the central nervous system (CNS) leading to sustained functional improvement is of paramount importance, given the personal suffering and cost associated with post-stroke disability (Ma et al., 2014). In addition to rehabilitation therapies to retrain degraded motor skills, animal and human studies have tried to augment recovery with neuropharmacologic interventions. Unfortunately, few if any have had a notable effect in patients or have come into routine use (Martinsson et al., 2007; Chollet et al., 2011; Cramer, 2015; Simpson et al., 2015). Methods to screen drugs based on their presumed mechanism of action on plasticity in human motor systems could speed translation to patients. However, there is currently no accepted method in stroke patients for evaluating the potential effectiveness or individual responsiveness to putative “plasticity enhancing” drugs in an efficient, low-cost, cross-sectional manner, in order to establish target engagement in humans and to avoid the extensive time and cost of protracted clinical trials.

Paired associative stimulation (PAS) is a safe, painless, and non-invasive technique known to result in short-term modulation of corticospinal excitability in the adult human motor system, lasting ∼90 min (Stefan et al., 2000; Wolters et al., 2003). Post-PAS excitability enhancement has been considered an LTP-like response thought to relate to transient changes in synaptic efficacy in the glutamatergic system at the *N*-methyl-D-aspartate (NMDA) receptor, since both human NMDA receptor deficiency (Volz et al., 2016) and pharmacological manipulation with dextromethorphan (Stefan et al., 2002) can block the effect. While PAS has been explored as a potential therapeutic intervention in patients with residual motor deficits after stroke (Jayaram and Stinear, 2008; Castel-Lacanal et al., 2009), it has not previously been investigated for its potential use as an assay of motor system plasticity in this context. Prior studies have suggested that motor practice and PAS share the same neuronal substrates, modulating LTP and LTD-like plasticity in the human motor system (Ziemann et al., 2004; Jung and Ziemann, 2009); therefore, as an established non-invasive human neuromodulation method (Suppa et al., 2017), we reasoned that PAS would be a suitable assay in the present study to examine the effect of a drug on motor system plasticity.

Here, we examine the effect of memantine, a drug used for treatment of Alzheimer’s disease, on the PAS response in patients with chronic stroke. Memantine is described pharmacologically as a low affinity, voltage dependent, non-competitive, NMDA antagonist (Rogawski and Wenk, 2003). At high concentrations, like other NMDA-R antagonists, it can inhibit synaptic plasticity. At lower, clinically relevant concentrations, memantine can, under some circumstances, promote synaptic plasticity by selectively inhibiting extra-synaptic glutamate receptor activity while sparing normal synaptic transmission, and hence may have clinical utility for rehabilitation (Xia et al., 2010). Interest in specifically using the drug for its interaction with stroke pathophysiology stems from animal models of both prevention (Trotman et al., 2015), in which pre-conditioning reduced infarct size, as well as for functional recovery, in which chronic oral administration starting >2 h post-stroke resulted in improved function through a non-neuroprotective mechanism (López-Valdés et al., 2014). In humans, memantine taken over multiple days has been used to demonstrate that the NMDA receptor is implicated in specific transcranial magnetic paired-pulse measures (Schwenkreis et al., 1999), and short-term training-induced motor map reorganization (Schwenkreis et al., 2005). In studies of neuromodulation, memantine blocked the facilitatory effect of intermittent theta-burst stimulation (iTBS) (Huang et al., 2007). Similarly, LTP-like plasticity induced by associative pairing of painful laser stimuli and TMS over primary motor cortex (M1) can also be blocked by memantine (Suppa et al., 2013). The effects of memantine on the PAS response have not yet been demonstrated, including examination of potential dose-response effects, which would be important for the potential clinical application of memantine for stroke recovery.

In our study, we set out to determine whether PAS might be a useful tool to probe the potential for plasticity after stroke in persons with chronic hemiparesis and apply PAS as an assay to look at drug effects on motor system plasticity using memantine. We hypothesized that (a) PAS would enhance corticospinal excitability in the contralesional hemisphere of stroke patients, and that (b) since PAS-induced plasticity is thought to involve a short-term change in glutamatergic synaptic efficacy, memantine would have a dose-dependent effect on PAS response. We predicted that at low doses, memantine would enhance PAS-induced plasticity through selective blockade of extrasynaptic NMDA receptors, whereas higher doses would inhibit PAS-induced plasticity.

## Materials and Methods

### Participants

Participants enrolled in the study were aged ≥18 years, with unilateral ischemic stroke at least 6 months prior, and upper limb paresis (≥7 points on the Fugl-Meyer impairment scale). Exclusion criteria were as follows: contraindications to transcranial magnetic stimulation (TMS; seizure/epilepsy history, pacemaker/other electro-sensitive implants), medications or conditions that affect the metabolism of memantine, concurrent pregnancy, hemorrhagic stroke, prior stroke, and co-existing neurological disorders. Written informed consent was obtained from all participants. The study had approval from the Institutional Review Board of the Burke Rehabilitation Center.

### Study Design and Drug Administration

The purpose of this experiment was to explore the potential of PAS to serve as a screening method for drugs of interest in neurorehabilitation. We used a within-subject repeated measures design to examine the effect of PAS in the contralesional hemisphere of hemiparetic persons with chronic stroke, and to test for a dose-effect of memantine on PAS response. Given the variable nature of MEPs and inter-individual responsiveness to neuromodulatory protocols, such a design is appropriate for this study. Participants attended over 5 weeks, including a screening visit and baseline assessment of clinical function and response to PAS. After the first PAS assessment, participants were given 5 mg of memantine, administered once daily for 1 week. The dosage of memantine was increased every 7 days by 5 mg to: 10, 15, and 20 mg on the 5th week. The dose of memantine was increased incrementally every week to assess for potential side effects with increasing doses. PAS response was reassessed weekly a day prior to the next dosing schedule, for a total of 5 PAS sessions per participant. The criterion measure for within session effects of PAS was MEP amplitude.

### Transcranial Magnetic Stimulation and Electromyography

Participants were seated reclined with arms relaxed and supported by a pillow, with surface EMG electrodes (Biometrics Ltd., SX230 1000× gain) over the first dorsal interosseous (FDI) muscle bilaterally, and ground electrode over the ulnar styloid. Via a head stage (Biometrics Gwen+ NPII 7 Hz 8 Channel), EMG was digitized at 5 kHz (Cambridge Electronic Design, Expansion ADC12/CED Micro1401 MKII), and band-pass filtered (20–1000 Hz; Spike 2, V7.15 CED). A Lycra cap with a 1 × 1 cm coordinate system was centered over the vertex (intersection of mid-point between nasion-inion and inter-aural lines). TMS was administered over the contralesional hemisphere, with the coil (Magstim Figure-8, double 70 mm) handle posterior and rotated laterally 45° from the midline. All TMS was performed with Magstim 200^2^ BiStim^2^ (Model 3010-00). The optimal site for FDI was explored with three to four suprathreshold pulses at each grid position commencing at C3/C4.

At each visit, motor threshold (MT) was calculated at rest, using the MT assessment tool (Awiszus, 2003). For baseline MEP assessment, stimulus intensity was set as follows: steps of 5% maximal stimulator output (MSO) commencing at 120% MT, 3 pulses per intensity, ceasing when MEP peak-to-peak amplitude increase reached a plateau (MEP max). The MTAT tool was then used to adjust the stimulator intensity to obtain an MEP amplitude representative of half of the participant’s MEP max. Twelve TMS pulses were delivered for each resting collection (pre/post-PAS) at ∼5–10 s intervals between pulses over 1–2 min. Resting motor threshold (RMT) and TMS intensity are reported as percentage of maximum stimulator output (%MSO) hereafter.

### Paired Associative Stimulation Intervention

Ulnar nerve electrical stimulation (DS7AH Digitimer Ltd., United Kingdom) was delivered with surface electrodes (8 mm diameter, 30 mm apart; Signa^®^ gel) positioned 3 cm above the palmar wrist crease of the unaffected arm, with cathode proximal (single 200 μs rectangular pulses). Perceptual threshold was defined as the minimum perceivable stimulator intensity (mA).

The PAS protocol approximated that described by Player et al. (2012), targeting the contralesional hemisphere, comprising 200 pairs of stimuli (TMS and ulnar nerve) given at 0.25 Hz over 13 min (Ziemann et al., 2004). In each pair, ulnar nerve stimulation (300% of perceptual threshold) preceded the TMS pulse (130% MT) by 25 ms. Baseline MEPs were collected immediately prior to the PAS intervention, followed by an immediate repeat of baseline at time 0 post, then every 10 min with a final recording at 60 min.

### Clinical, Functional, and Neurophysiological Evaluation Across Weeks

An upper limb clinical impairment measure (Fugl-Meyer scale, FM) and a functional assessment (Wolf motor function test) were performed at the start and end of the study, in order to capture clinical status post 4 weeks of incremental memantine administration and PAS, relative to baseline.

Participants performed a weekly dexterity test (9-hole peg test, single trial, total time, seconds) and a strength test (maximum grip, mean of 3 trials, lbs.), with both the unaffected and the affected hand, so as to capture any potential effects of brain stimulation and drug on clinical function. Dexterity and strength tests were performed at the scheduled clinic visit prior to the PAS intervention. No upper extremity rehabilitation was provided.

RMT and sensory perceptual threshold were assessed weekly to determine stimulator intensity for the PAS protocol.

### Data Analysis

#### Motor Evoked Potential Processing

MEPs were visually inspected offline and analyzed for peak-to-peak amplitude using Spike 2 software (Cambridge Electronic Design, Cambridge, United Kingdom). The first two pulses from each collection were routinely excluded, as were MEPs with background EMG >25 μV within 100 ms prior to the TMS stimulus. The median MEP amplitude was averaged across participants at each time point for the group analysis, repeated for each dose of drug.

#### Statistical Analyses

We compared the mean MEP amplitude over the 1st hour post-PAS intervention versus MEP amplitude immediately before intervention (baseline), with a non-parametric rank-sum test for paired differences, and a one-sided *p*-value of 0.05 testing for an increase in amplitude with PAS, for the off-drug condition and for each memantine dose.

We also explored each 10-min interval of the 1st hour post-intervention, comparing mean absolute change in MEP amplitude, and the proportion of participants who increased, using two-sided exact binomial tests. This was again repeated for each memantine dose.

To assess potential long-term effects of PAS or memantine, we compared clinical measures at the start of the study to the same assessments 1 week after the last dose, using the non-parametric rank-sum test for paired differences and a one-sided *p*-value of 0.05.

#### Exploratory Responder Analysis

In secondary analyses, we stratified participants into PAS responders and non-responders according to their response to PAS in the baseline session (off-drug), as per Müller-Dahlhaus et al. (2015) and Strube et al. (2015), where responders were defined as subjects exhibiting a post-intervention MEP amplitude increase of >10% from baseline. We evaluated clinical and neurophysiological features that were associated with having a response to PAS. Interhemispheric difference in corticospinal excitability was calculated by subtracting the ipsilesional hemisphere RMT value from the contralesional hemisphere RMT value for each subject. A RMT value of 100 was assigned for cases in which RMT was unable to be derived from the ipsilesional hemisphere (Stinear et al., 2015). We further evaluated the interaction between baseline responder status and MEP amplitude increase post-PAS with the different doses of memantine.

## Results

### Participant Clinical Characteristics

**FIGURE 1:**
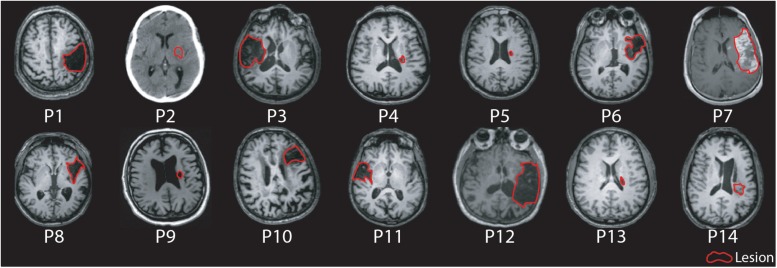
Axial MR/CT images for individual patients illustrating the stroke lesion. Images are displayed in radiological convention. Images are labeled by participant number.

Fourteen participants with chronic hemiparetic stroke were enrolled and completed the study (age range 48–91 years, four female/ten male, 6–161 months post-ischemic stroke; see Table 1 for baseline clinical and neurophysiological characteristics at study entry, and Figure 1 for imaging of lesion location). Upper limb Fugl-Meyer impairment score at study entry ranged from 7 to 60 points (mean 32.7, out of a maximum possible 66 points). PAS and memantine appeared to be safe and well tolerated by all participants; there were no adverse events related to the study medication or PAS.

**TABLE 1:** Participant characteristics at study entry.

**ID**	**Time since onset (months)**	**FM-UE**	**WMFT (s)**	**rMT (%MSO)**		**Medication**
				**U**	**A**	
P1	18	7	1613.9	47	NR	Insulin, albuterol, tamsulosin
P2	94	19	1367.1	53	NR	ASA, simvastatin, levetiracetam, levothyroxine, alendronic acid
P3	28	14	1802.6	54	NR	ASA, escitalopram
P4	100	30	674	43	NR	ASA, lisinopril
P5	27	48	54.3	48	NR	Losartan, nebivolol, atorvastatin, sertraline
P6	46	20	1452.5	55	NR	ASA, atorvastatin, escitalopram
P7	64	7	1448.1	42	NR	ASA, amlodipine, simvastatin, tamsulosin, escitalopram
P8	161	50	35.3	53	61	Warfarin, metoprolol, lisinopril, digoxin, simvastatin, metformin
P9	80	34	56.7	49	51	ASA, lisinopril, simvastatin, tamsulosin, dutasteride
P10	35	53	30.6	43	48	ASA, carvedilol, quinapril, HCTZ, atorvastatin
P11	28	48	36.3	33	34	Apixaban, sotalol, atorvastatin, pantoprazole, citalopram, gabapentin, trazodone
P12	149	24	1565.7	49	46	Clopidogrel, atorvastatin, oxybutynin, hydrocodone
P13	6	60	27.9	44	64	ASA, atorvastatin, fluoxetine, baclofen
P14	20	44	65.6	35	53	ASA, atenolol, amlodipine-benazepril, atorvastatin, duloxetine, gabapentin

*FM-UE, Fugl-Meyer Upper Extremity; WMFT, Wolf Motor Function Test; rMT, resting Motor Threshold; MSO, Maximum Stimulator Output; U, Unaffected Hemisphere; A, Affected Hemisphere; NR, No Response; ASA, Aspirin; HCTZ, Hydrochlorothiazide.*

### Within-Session PAS Effects

**FIGURE 2:**
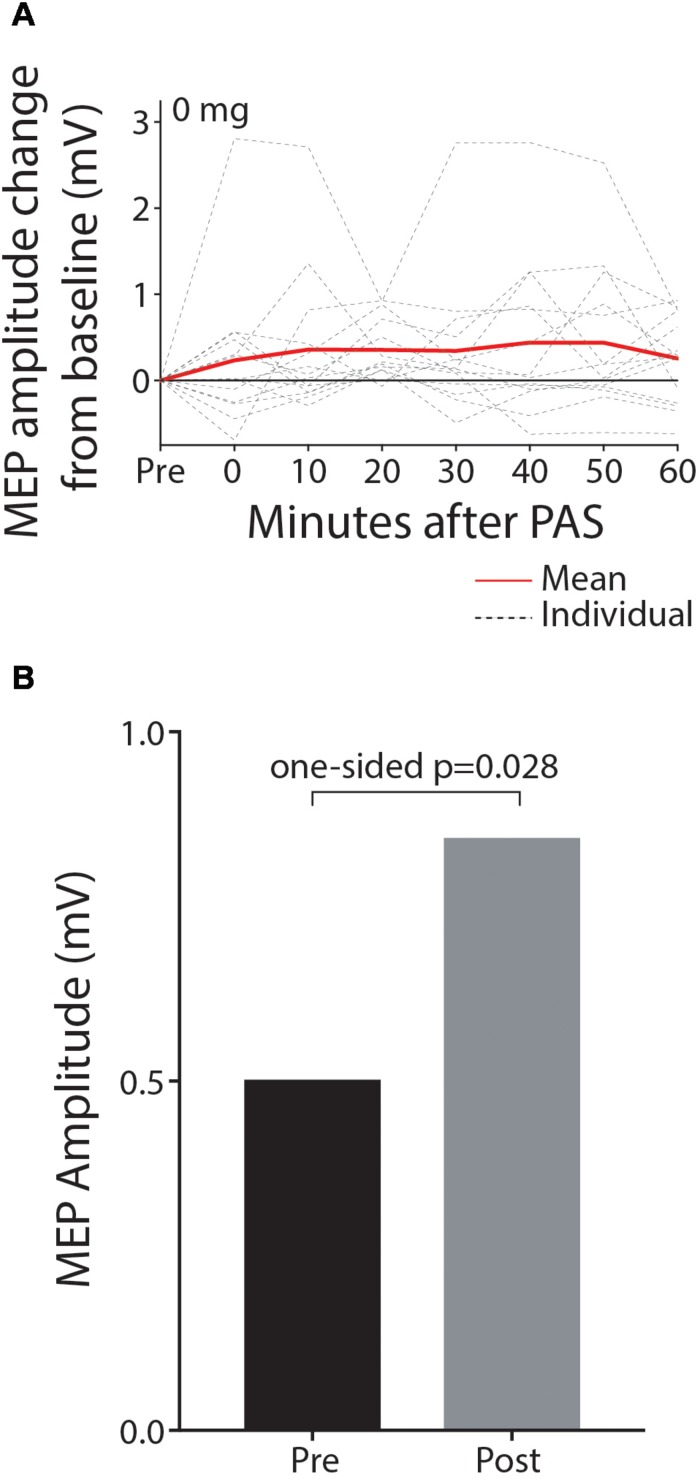
MEP amplitude is significantly increased post-PAS intervention relative to pre-PAS amplitude in the baseline off-drug session. Data are shown depicting MEP amplitude change from baseline for individual time-points post-PAS **(A)** and mean MEP amplitude for pre and the hour post-PAS **(B)**.

**FIGURE 3:**
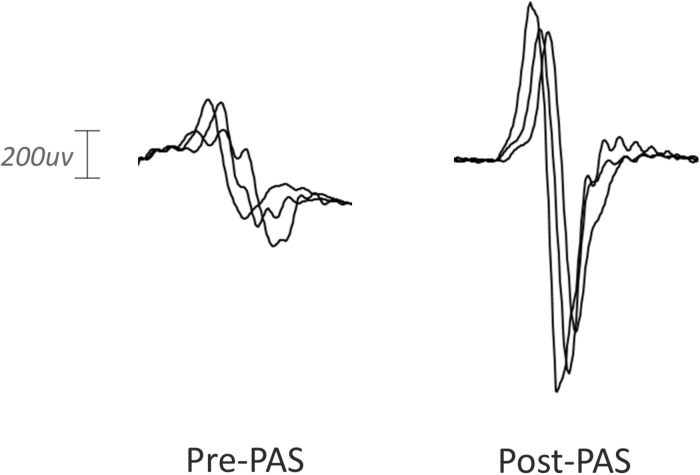
Representative MEP waveforms from a single participant pre and post-PAS intervention. Peak-to-peak MEP amplitude was enhanced by PAS in the baseline session.

Paired associative stimulation successfully increased corticospinal excitability in the contralesional hemisphere in the baseline session without memantine. The mean MEP amplitude was significantly higher after PAS intervention as shown in Figures 2A,B (0.504 mV pre, 0.851 mV post, 0.346 mV mean increase; 90% CI 0.055–0.638; one-sided *p* = 0.028, median increase 0.364, one-sided *p* = 0.045; sample waveforms are shown in Figure 3). The numerically largest increase in MEP amplitude was seen 20 min post-intervention (Figure 2A), where 85.7% of participants had increased MEP amplitude (one-sided *p* = 0.013), and 78.5% had increased by at least 10% (one-sided *p* < 0.001).

**FIGURE 4:**
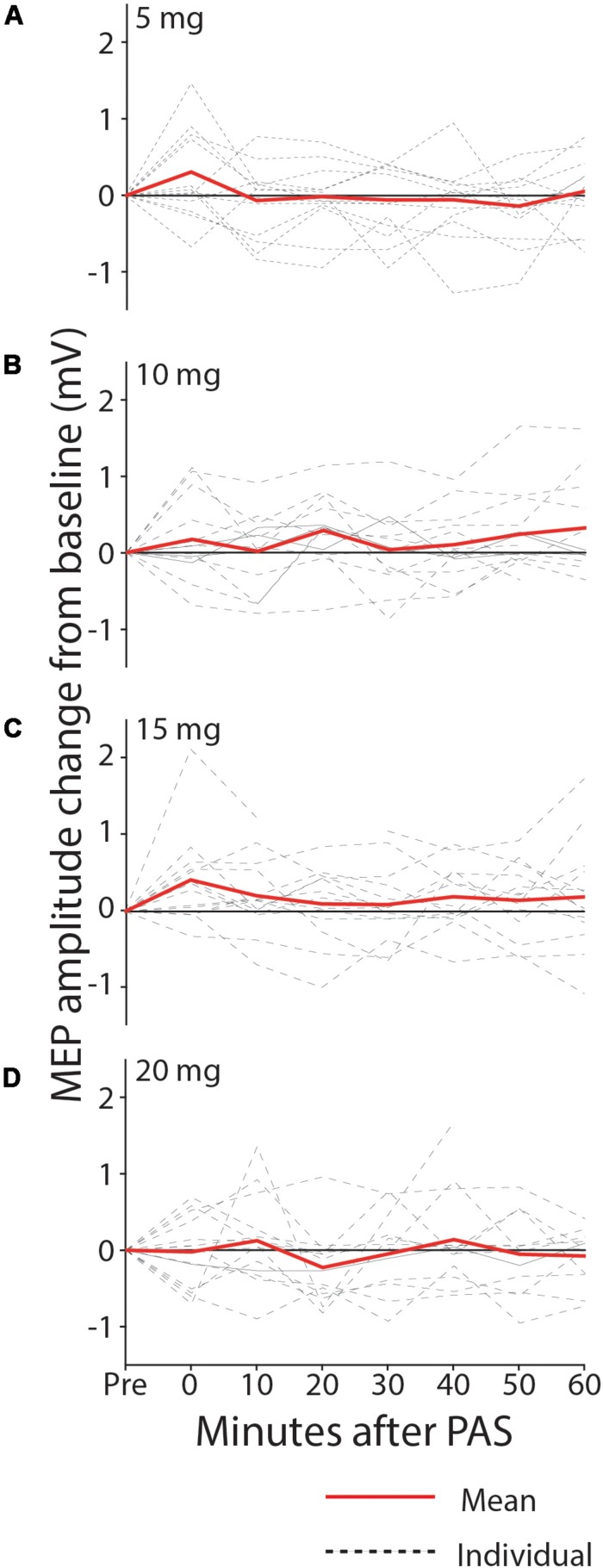
MEP change from baseline after PAS with 5 mg **(A)**, 10 mg **(B)**, 15 mg **(C)**, and 20 mg **(D)** memantine. Group MEP amplitude over the 60 min following PAS was not significantly increased from baseline, with any dose of memantine.

With all doses of memantine (5–20 mg), there was no significant increase in MEP amplitude after PAS at the group level, suggesting that memantine blocked synaptic plasticity at the range of doses tested, contrary to our prediction that low doses would enhance PAS-induced plasticity. In the 5 mg condition^1^ (Figure 4A), MEP amplitude was not increased from baseline following PAS intervention (0.589 mV pre, 0.597 mV post, 0.009 mV mean increase; 90% CI −0.174–0.191; *p* = 0.467; median increase 0.0001, *p* = 0.620). With 10 mg memantine (Figure 4B), MEP amplitude was again not found to increase from baseline (0.622 mV pre, 0.728 mV post, 0.106 mV mean increase; 90% CI −0.046–0.258; *p* = 0.119; median increase 0.078, *p* = 0.195). Similarly, MEP amplitude was not increased from baseline with 15 mg memantine (Figure 4C; 0.594 mV pre, 0.794 mV post, 0.200 mV mean increase; 90% CI -0.014-0.414; *p* = 0.061; median increase 0.164, *p* = 0.077) or with 20 mg memantine (Figure 4D; 0.642 mV pre, 0.642 mV post, 0.001 mV mean increase; 90% CI -0.181-0.181; *p* = 0.499; median increase 0.001, *p* = 0.548).

### Clinical, Functional, and Neurophysiological Assessment Across Weeks

We did not observe significant changes between the first and last week of intervention in RMT in the ipsilesional hemisphere (*n* = 7 evaluable, average change-0.71%MSO [90%CI –2.64, 1.22], *p* = 0.750) nor in the contralesional hemisphere (*n* = 14, −0.61[3.27, 2.04], *p* = 0.656). We also observed no significant changes in clinical measures of grip strength (1.43lbs [−2.59, 5.44], *p* = 0.270; affected *n* = 11, 4.54 [1-0.76, 10.85], *p* = 0.11 unaffected, *n* = 14), dexterity as measured with the 9 hole peg test (−0.61s [−3.27, 2.04], *p* = 0.656 affected *n* = 6, 10.93 [−32.07, 53.94], *p* = 0.315 unaffected *n* = 14), or sensory perceptual threshold (0.350 mA [−0.375, 1.075], *p* = 0.204).

### Exploratory Responder Analysis

**FIGURE 5:**
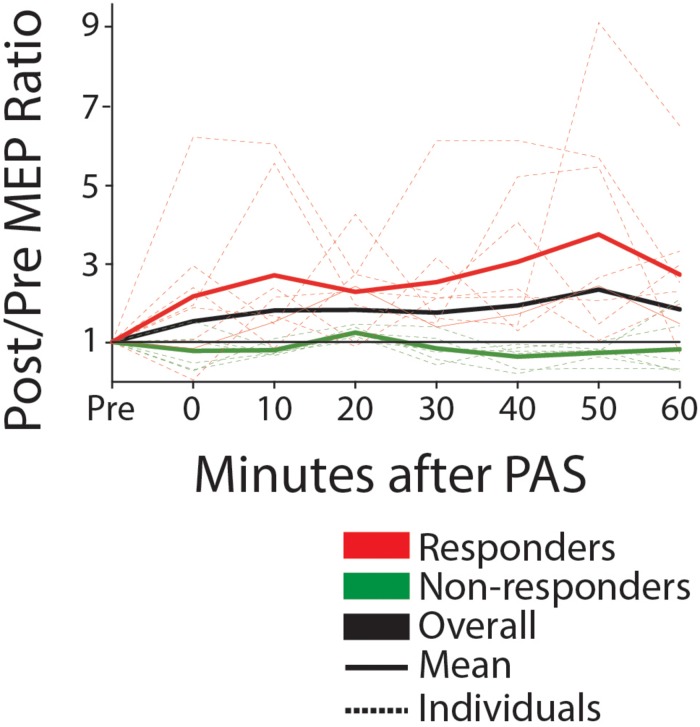
MEP amplitude pre and post-PAS (0–60 min) for responders (mean post >10% mean pre) and non-responders, off-drug (ratio post/pre). Solid lines show group mean, dotted show individual data. Black line is the mean for the entire group. We note that approximately half of participants are responders, with a mean increase of greater than 200% post versus pre.

Eight of fourteen participants were classified as responders to PAS, defined as subjects exhibiting a post-PAS MEP amplitude increase of >10%. Responders had a mean post-PAS increase of >200% in MEP amplitude (post/pre ratio, 2.23 [1.82; 3.33] Figure 5). The responders were clinically more severe as shown by increased time on the Wolf Motor Function Test (responder, mean = 1450 ± 719 SD sec; non-responder, 46 ± 541, *p* = 0.028), lower grip strength (responder, mean = 17.5 ± 13.3 SD lbs.; non-responder, 53 ± 27.5, *p* = 0.01), and TMS measures (six of eight responders had no detectable MEP on the affected side; versus one of six non-responders). Other clinical (FM) and neurophysiological (RMT difference) measures were in the same direction of greater severity, but were not statistically significant. Baseline corticospinal excitability (RMT) in the contralesional (intervention) hemisphere was comparable between responders and non-responders (mean responder, 46.4 ± 7.1; mean non-responder, 46.2 ± 6.9; median responder, 47.5 [42.8; 50.2]; median non-responder, 46.5 [43.2; 52.0] *p* = 1.00). There was no significant interaction between responder status and the mean increase in MEP amplitude post-PAS with any dose of memantine (see Supplementary Table 1) (5 mg condition; *F* = 0.450, *p* = 0.823, 10 mg condition; *F* = 1.681, *p* = 0.128, 15 mg condition; *F* = 2.296, *p* = 0.190, 20 mg condition; *F* = 1.297, *p* = 0.295).

## Discussion

In the present study, we examined whether PAS MEP-potentiation could be a surrogate biomarker of the likelihood that a particular medication at an optimal dose may augment plasticity. If proven viable, this methodology would be invaluable to the field, as there is currently no accepted method in stroke patients for evaluating the potential effectiveness or individual responsiveness to varying doses of putative ‘plasticity enhancing’ drugs in an efficient, low-cost, cross-sectional manner. This would be the first step toward trials testing therapeutic drugs in combination with training aimed at improving motor skills after disabling stroke. PAS has received interest for improving motor function post-stroke (Jayaram and Stinear, 2008; Castel-Lacanal et al., 2009), however, response to a single application as a window into the capacity for human brain plasticity has not been evaluated in this context. We showed that without memantine, PAS resulted in an after-effect of heightened excitability (MEP amplitude increase) consistent with the literature in healthy adults (Stefan et al., 2000; Player et al., 2012). When participants were assessed following 1 week of memantine administration, at every dose evaluated (5–20 mg), excitability remained at baseline levels post-intervention, indicating that the PAS-induced excitability enhancement was blocked by memantine as assessed at steady-state. Although the predictive value of PAS for drug-induced functional recovery post-stroke has yet to be established, our data suggest that, while clinical doses of memantine can penetrate into the CNS to affect function, we found no evidence that it enhances plasticity in the contralesional hemisphere in those with chronic hemiparetic stroke.

### Effect of PAS Off-Drug

MEP amplitude was raised over the 60 min post-intervention relative to baseline, with the most consistent effect across participants occurring 20 min post-intervention. Delayed peak after-effects have been previously reported (Müller-Dahlhaus et al., 2010). Variation in both the time-course and magnitude of the PAS response could be influenced by factors including disease state, lesion location, and concurrent medications. Stroke patients typically have comorbidities (Ostwald et al., 2006), managed with medications that may affect the response to TMS (Siebner and Ziemann, 2007; Ziemann et al., 2014). In our sample, thirteen of the fourteen participants were taking drugs known to affect the response to brain stimulation, or medications that have a similar neuro-pharmacological mechanism of action to such drugs, including SSRI for the treatment of depression (Siebner and Ziemann, 2007), and anti-spasticity medication such as baclofen (Ziemann et al., 2014). PAS response may also be influenced by genotype-related differences such as BDNF and COMT polymorphism interactions (Witte et al., 2012). Experimentally, we aimed to reduce predicted variability in the quantified response by (a) using a greater sample of MEPs post-intervention, which we have previously shown can increase the sensitivity for detecting MEP amplitude change (Bastani and Jaberzadeh, 2012), and (b) by adjusting stimulus intensity to half-maximum MEP amplitude at baseline, thus accounting for individual differences and potential ceiling effects in contralesional hemisphere excitability. Although inter-subject response variation is characteristic in non-invasive neuromodulation (Wiethoff et al., 2014; Murase et al., 2015; Schambra et al., 2015), our observed variation in MEP amplitude increase post-intervention may suggest an alternative methodological approach for PAS application in post-stroke drug dosing studies (responder analysis; see below).

### Drug Selection

Our selection of the drug memantine was in part based on prior studies in hippocampal microcultures by Lipton et al., (Xia et al., 2010). We hypothesized that low doses of the clinically approved, use-dependent glutamate antagonist, memantine (5–10 mg), would enhance PAS-induced excitability in the contralesional hemisphere via its ability to selectively block extrasynaptic glutamate receptors, while leaving synaptic glutamate receptors unperturbed. Extrasynaptic glutamate activity has been hypothesized to block plasticity-associated gene expression mediated via activation of synaptic glutamate (Hardingham and Bading, 2010). Contrary to our hypothesis, the current study showed that PAS-induced neuromodulation in chronic stroke was abrogated at all doses of memantine. This finding also favors a synaptic LTP-like mechanism for post-PAS excitability enhancement, and is consistent with a separate study using intermittent theta burst stimulation (iTBS) in healthy human subjects, where memantine blocked the excitability enhancing effect of iTBS (Huang et al., 2007). It remains possible that synaptic plasticity would be enhanced with memantine at lower doses (<5 mg) than those tested in this study. Additionally, Ziemann et al. (2004) demonstrated that LTP-dependent motor learning can block subsequent induction of LTP via PAS due to homeostatic metaplasticity; therefore, LTP-like plasticity induced by memantine could have blocked the effects of further LTP induction via PAS. This can be tested in future experiments using a LTD-like PAS protocol.

### Stability of Electrophysiological and Clinical Measures

We did not observe significant changes in RMT, sensory perception, or motor function across the study period, indicating that both escalating drug dose, as well as weekly PAS intervention, had no cumulative effect on these measures. RMT, the individually adjusted minimum stimulator output required to elicit consistent MEPs, did not change regardless of the memantine dose, indicating high test–retest reliability and no effect of drug on this measure. RMT is thought to represent neuronal membrane excitability, since it is increased by drugs that block voltage-gated sodium channels such as carbamazepine (Ziemann et al., 1996), and is not affected by drugs influencing glutamatergic synapse activity such as riluzole (Liepert et al., 1997). Stable RMT across the study period was important experimentally, as threshold-adjusted PAS stimulus intensity remained constant, providing validity for between-dose comparisons.

Somatosensory perceptual threshold for peripheral nerve stimulation did not change across the 5 weeks of repeated PAS and daily memantine administration, indicating a neutral effect of memantine on sensory-afferent awareness and precision in detection of peripheral stimuli. There was no change in measures of impairment (Fugl-Meyer scale) or function (Wolf motor function test), thus clinical motor status remained stable over the study period. The weekly dexterity and strength measures also did not significantly vary. Taken together, these findings indicate that while PAS plasticity is not observed in the presence of memantine, there was no appreciable disruption to functional performance in either the PAS-targeted hand (unaffected) or the affected hand of chronic stroke patients, associated with weekly escalating dose of memantine, and repeated exposure to PAS.

### Exploratory Responder Analysis

In the present study, approximately 57% of participants were classified as responders to PAS based on their response in the off-drug condition, which is consistent with other studies, at slightly above 50% (Müller-Dahlhaus et al., 2008; Lahr et al., 2016). We found that stroke symptom severity (measured by grip strength and WMFT) and absence of MEP in the lesioned hemisphere were predictors of response to PAS in the contralesional hemisphere, where baseline excitability was comparable across participants. This pattern of clinical and neurophysiological features associated with responsiveness to PAS is consistent with results described by Ferris et al. (2018). Plasticity induction occurred more readily in the contralesional hemisphere of more severe stroke patients, which could plausibly be due to a greater propensity for hyperexcitability in the contralesional hemisphere. We note that the degree of MEP amplitude increase post-intervention in our responder population (mean 100–300%) was higher than previous reports in the literature for healthy subjects, which ranges from 20 to 78% (Ziemann et al., 2004; Fratello et al., 2006; Müller-Dahlhaus et al., 2008; Ceccanti et al., 2018). However, the relatively scarce studies of PAS response in stroke subjects describe a wider range of response variability, with MEP facilitation ranging from 40% to over 300% (Castel-Lacanal et al., 2009; Palmer et al., 2018). We observed that five of the eight responders in our population were taking SSRI medications, which have been reported to enhance single pulse MEP amplitude (Siebner and Ziemann, 2007) as well as augment PAS effects (Batsikadze et al., 2013).

The effect of memantine was observed in both the PAS responder (more clinically affected) and non-responder (less clinically affected) groups. However, if a plasticity-enhancing drug could be beneficial for a subgroup of patients who are more severely affected but more susceptible to plasticity, this could have important implications in stroke recovery and improve precision in therapeutic prescription.

### Limitations and Future Directions

As we tested the contralesional hemisphere, it is possible that a differential effect of memantine occurs in the ipsilesional hemisphere. We pursued to study the contralesional hemisphere due to concerns of reliability and validity of the PAS protocol in the ipsilesional hemisphere, as per Ferris et al. (2018). Reduced efferent and/or afferent conduction, or absent response completely, could impact the effectiveness of PAS, which is considered to be dependent on a narrow window of arrival of the peripheral stimulus afferent volley with the transcranial motor cortex stimulation. Our current findings suggest that the contralesional hemisphere is a suitable target for assessing PAS-induced plasticity, since the RMT was not reduced (hyper-excitable) in relation to our prior studies in chronic stroke (Schambra et al., 2015), and was comparable in both responder and non-responder participants.

While our sample size may not be large, one must appreciate the extent of the investigation that was conducted. This study comprised a total of seventy PAS sessions, each involving the collection of 96 MEPs. This represents one of the largest trials utilizing PAS in the stroke population, with the exception of a single study examining PAS as a potential therapeutic intervention for stroke recovery (Tarri et al., 2018). An aim of this investigation was to explore PAS as a potential screening method for drugs of interest in neurorehabilitation, prior to investing additional time and resources in a more extensive trial. Our results neither support nor preclude the use of PAS as an effective screening tool for drug effects on plasticity. Further investigation is warranted to determine whether the observations gleaned from this trial are the results of homeostatic metaplasticity.

Since PAS-induced excitability modulation is thought involve the NMDA receptor, as supported by our results with memantine, future studies in a stroke population could test the effects of other commonly used agents acting on the NMDA receptor, specifically with drugs that enhance activation of the NMDA receptor complex such as D-cycloserine (Lanthorn, 1994), or a newer generation of memantine, nitromemantine (Takahashi et al., 2015). The specific strategy advocated here was to determine the optimal dose for stimulating plasticity in the human brain by using PAS potentiation as a surrogate marker of enhanced plasticity. For example, PAS may have been useful to identify whether the dose of D-cycloserine employed was optimal before the longitudinal intervention trial in stroke rehabilitation (Butler et al., 2015) which did not show a drug benefit.

Here, we studied a chronic stroke population, though our approach could be applied in the early post-stroke period to examine impact on plasticity with emerging neuroprotective/neuroplasticity agents such as NA-1 (Tat-NR2B9c), an inhibitor of postsynaptic density-95 protein (Hill et al., 2012; Dobkin, 2017), or maraviroc, a CCR5 antagonist (Sorce et al., 2010). The use of PAS as plasticity marker should be contingent upon the reproducibility of studies of PAS in stroke. We recommend first assessing response to PAS and reproducibility prior to an individual entering the drug arm of a study.

Finally, while our finding of PAS corticospinal excitability enhancement serves as a motor domain specific effect that might be useful in post-stroke hemiparesis, PAS could plausibly be a surrogate for drug-effects on plasticity in non-motor domains.

## Conclusion

We report the first study using non-invasive neuromodulation to assay plasticity in the post-stroke human motor system for the purpose of testing a clinically used neuroplasticity drug with potential utility in neurorehabilitation. We showed that PAS is effective for increasing corticospinal output in the contralesional hemisphere in chronic post-stroke upper limb paresis, and may serve as an assay for CNS target engagement for drugs of interest in post-stroke motor recovery. Applying this model to test a candidate drug, memantine, we found that memantine blocked PAS-induced plasticity, indicating CNS target engagement, but our findings do not support the use of memantine at the doses tested for enhancing motor system plasticity for rehabilitation.

## Ethics Statement

The study had approval from the Institutional Review Board of the Burke Rehabilitation Center. All procedures performed in studies involving human participants were in accordance with the ethical standards of the institutional and/or national research committee and with the 1964 Helsinki declaration and its later amendments or comparable ethical standards. Informed consent was obtained from all individual participants included in the study.

## Author Contributions

JS: writing, data interpretation, data analysis, data collection, protocol revisions, figures, and literature search. MC: writing, data collection, and literature search. KT: data collection and figures. AC: writing, data analysis, data collection, protocol revisions, figures, and literature search. CO and LG: writing, statistical analysis, and figures. PF: physician, data interpretation, and literature search. RR: study design, writing, data interpretation, and literature search. TK: writing, data interpretation, and literature search. MI: study design, writing, data interpretation, protocol revisions, figures, literature search, and figures. BD and AW: study design, writing, data interpretation, protocol revisions, and literature search. DE: study design, writing, data interpretation, data analysis, protocol revisions, figures, and literature search.

## Conflict of Interest Statement

The authors declare that the research was conducted in the absence of any commercial or financial relationships that could be construed as a potential conflict of interest.

## Footnotes

1 We conservatively removed one subject from analysis at 5 mg where the median MEP value at baseline was <50 μV, who therefore showed a disproportionately high response at over 15-fold increase.

## References

[B1] Awiszus F. (2003). TMS and threshold hunting. *Suppl. Clin. Neurophysiol.* 56 13–23. 14677378 10.1016/s1567-424x(09)70205-3

[B2] Bastani A. Jaberzadeh S. (2012). A higher number of TMS-Elicited MEP from a combined hotspot improves intra- and inter-session reliability of the upper limb muscles in healthy individuals. *PLoS One* 7:e47582. 10.1371/journal.pone.0047582 23077645 PMC3471890

[B3] Batsikadze G. Paulus W. Kuo M.-F. Nitsche M. A. (2013). Effect of serotonin on paired associative stimulation-induced plasticity in the human motor cortex. *Neuropsychopharmacology* 38 2260–2267. 10.1038/npp.2013.127 23680943 PMC3773677

[B4] Butler A. J. Kallos J. Housley S. N. Laplaca M. C. Traynelis S. F. Wolf S. L. (2015). ). Randomized, placebo-controlled, double-blind pilot study of D-Cycloserine in chronic stroke. *Rehabil. Res. Pract.* 2015:534239. 10.1155/2015/534239 26587287 PMC4637506

[B5] Castel-Lacanal E. Marque P. Tardy J. Boissezon X. D. Guiraud V. Chollet F. (2009). Induction of cortical plastic changes in wrist muscles by paired associative stimulation in the recovery phase of stroke patients. *Neurorehabil. Neural Repair* 23 366–372. 10.1177/1545968308322841 19060132

[B6] Ceccanti M. Onesti E. Rubino A. Cambieri C. Tartaglia G. Miscioscia A. (2018). Modulation of human corticospinal excitability by paired associative stimulation in patients with amyotrophic lateral sclerosis and effects of Riluzole. *Brain Stimul.* 11 775–781. 10.1016/j.brs.2018.02.007 29459142

[B7] Chollet F. Tardy J. Albucher J.-F. Thalamas C. Berard E. Lamy C. (2011). Fluoxetine for motor recovery after acute ischaemic stroke (FLAME): a randomised placebo-controlled trial. *Lancet Neurol.* 10 123–130. 10.1016/S1474-4422(10)70314-8 21216670

[B8] Cramer S. C. (2015). Drugs to enhance motor recovery after stroke. *Stroke J. Cereb. Circul.* 46 2998–3005. 10.1161/STROKEAHA.115.007433 PMC4589468 26265126

[B9] Dobkin B. (2017). ClinicalTrials.gov (Identifier NCT03172026). *Maraviroc to Augment Rehabilitation Outcomes After Stroke (MAROS).* Bethesda, MD: United States National Library of Medicine Available at: https://clinicaltrials.gov/ct2/show/NCT03172026

[B10] Ferris J. K. Neva J. L. Francisco B. A. Boyd L. A. (2018). Bilateral motor cortex plasticity in individuals with chronic stroke, induced by paired associative stimulation. *Neurorehabil. Neural Repair* 32 671–681. 10.1177/1545968318785043 29969936

[B11] Fratello F. Veniero D. Curcio G. Ferrara M. Marzano C. Moroni F. (2006). Modulation of corticospinal excitability by paired associative stimulation: reproducibility of effects and intraindividual reliability. *Clin. Neurophysiol.* 117 2667–2674. 17011821 10.1016/j.clinph.2006.07.315

[B12] Hardingham G. E. Bading H. (2010). Synaptic versus extrasynaptic NMDA receptor signalling: implications for neurodegenerative disorders. *Nat. Rev. Neurosci.* 11 682–696. 10.1038/nrn2911 20842175 PMC2948541

[B13] Hill M. D. Martin R. H. Mikulis D. Wong J. H. Silver F. L. Terbrugge K. G. (2012). Safety and efficacy of NA-1 in patients with iatrogenic stroke after endovascular aneurysm repair (ENACT): a phase 2, randomised, double-blind, placebo-controlled trial. *Lancet Neurol.* 11 942–950. 10.1016/S1474-4422(12)70225-9 23051991

[B14] Huang Y.-Z. Chen R.-S. Rothwell J. C. Wen H.-Y. (2007). The after-effect of human theta burst stimulation is NMDA receptor dependent. *Clin. Neurophysiol.* 118 1028–1032. 17368094 10.1016/j.clinph.2007.01.021

[B15] Jayaram G. Stinear J. W. (2008). Contralesional paired associative stimulation increases paretic lower limb motor excitability post-stroke. *Exp. Brain Res.* 185 563–570. 17973101 10.1007/s00221-007-1183-x

[B16] Jung P. Ziemann U. (2009). Homeostatic and nonhomeostatic modulation of learning in human motor cortex. *J. Neurosci.* 29 5597–5604. 10.1523/JNEUROSCI.0222-09.2009 19403826 PMC6665848

[B17] Lahr J. Paßmann S. List J. Vach W. Flöel A. Klöppel S. (2016). Effects of different analysis strategies on paired associative stimulation. a pooled data analysis from three research labs. *PLoS One* 11:e0154880. 10.1371/journal.pone.0154880 27144307 PMC4856316

[B18] Lanthorn T. H. (1994). D-Cycloserine: agonist turned antagonist. *Amino Acids* 6 247–260. 10.1007/BF00813745 24189733

[B19] Liepert J. Schwenkreis P. Tegenthoff M. Malin J.-P. (1997). The glutamate antagonist riluzole suppresses intracortical facilitation. *J. Neural. Transm.* 104 1207–1214. 9503266 10.1007/BF01294721

[B20] López-Valdés H. E. Clarkson A. N. Ao Y. Charles A. C. Carmichael S. T. Sofroniew M. V. (2014). Memantine enhances recovery from stroke. *Stroke J. Cereb. Circulat.* 45 2093–2100. 10.1161/STROKEAHA.113.004476 PMC4142682 24938836

[B21] Ma V. Chan L. Carruthers K. (2014). The incidence, prevalence, costs and impact on disability of common conditions requiring rehabilitation in the US: stroke, spinal cord injury, traumatic brain injury, multiple sclerosis, osteoarthritis, rheumatoid arthritis, limb loss, and back pain. *Arch. Phys. Med. Rehabil.* 95:986-995.e1. 10.1016/j.apmr.2013.10.032 PMC4180670 24462839

[B22] Martinsson L. Hårdemark H.-G. Eksborg S. (2007). Amphetamines for improving recovery after stroke. *Cochrane Database Syst. Rev.* CD002090. 10.1002/14651858.CD002090.pub2 17253474 PMC12278358

[B23] Müller-Dahlhaus F. Lücke C. Lu M.-K. Arai N. Fuhl A. Herrmann E. (2015). Augmenting LTP-Like Plasticity in Human Motor Cortex by Spaced Paired Associative Stimulation. *PLoS One* 10:e0131020. 10.1371/journal.pone.0131020 26110758 PMC4482149

[B24] Müller-Dahlhaus F. Ziemann U. Classen J. (2010). Plasticity resembling spike-timing dependent synaptic plasticity: the evidence in human cortex. *Front. Synaptic Neurosci.* 2:34. 10.3389/fnsyn.2010.00034 21423520 PMC3059695

[B25] Müller-Dahlhaus J. F. M. Orekhov Y. Liu Y. Ziemann U. (2008). Interindividual variability and age-dependency of motor cortical plasticity induced by paired associative stimulation. *Exp. Brain Res.* 187 467–475. 10.1007/s00221-008-1319-7 18320180

[B26] Murase N. Cengiz B. Rothwell J. C. (2015). Inter-individual variation in the after-effect of paired associative stimulation can be predicted from short-interval intracortical inhibition with the threshold tracking method. *Brain Stimul.* 8 105–113. 10.1016/j.brs.2014.09.010 25444589

[B27] Ostwald S. K. Wasserman J. Davis S. (2006). Medications, comorbidities, and medical complications in stroke survivors: the CAReS study. *Rehabil. Nurs.* 31 10–14. 16422039 10.1002/j.2048-7940.2006.tb00004.x PMC1405842

[B28] Palmer J. A. Wolf S. L. Borich M. R. (2018). Paired associative stimulation modulates corticomotor excitability in chronic stroke: a preliminary investigation. *Restor. Neurol. Neurosci.* 36 183–194. 10.3233/RNN-170785 29526858 PMC5870032

[B29] Player M. J. Taylor J. L. Alonzo A. Loo C. K. (2012). Paired associative stimulation increases motor cortex excitability more effectively than theta-burst stimulation. *Clin. Neurophysiol.* 123 2220–2226. 10.1016/j.clinph.2012.03.081 22608487

[B30] Rogawski M. A. Wenk G. L. (2003). The neuropharmacological basis for the use of memantine in the treatment of alzheimer’s disease. *CNS Drug Rev.* 9 275–308. 14530799 10.1111/j.1527-3458.2003.tb00254.x PMC6741669

[B31] Schambra H. M. Ogden R. T. Martínez-Hernández I. E. Lin X. Chang Y. B. Rahman A. (2015). The reliability of repeated TMS measures in older adults and in patients with subacute and chronic stroke. *Front. Cell. Neurosci.* 9:335. 10.3389/fncel.2015.00335 26388729 PMC4555014

[B32] Schwenkreis P. Witscher K. Janssen F. Addo A. Dertwinkel R. Zenz M. (1999). Influence of the N-methyl-D-aspartate antagonist memantine on human motor cortex excitability. *Neurosci. Lett.* 3 137–140. 10462113 10.1016/s0304-3940(99)00492-9

[B33] Schwenkreis P. Witscher K. Pleger B. Malin J.-P. Tegenthoff M. (2005). The NMDA antagonist memantine affects training induced motor cortex plasticity – a study using transcranial magnetic stimulation [ISRCTN65784760]. *BMC Neurosci.* 6:35. 10.1186/1471-2202-6-35. 15890074 PMC1134663

[B34] Siebner H. R. Ziemann U. (2007). *Das TMS-Buch: Handbuch Der Transkraniellen Magnetstimulation.* Berlin: Springer-Verlag.

[B35] Simpson D. Goldenberg J. Kasner S. (2015). Dalfampridine in chronic sensorimotor deficits after ischemic stroke: a proof of concept study. *J. Rehabil. Med.* 47 924–931. 10.2340/16501977-2033 26540083

[B36] Sorce S. Bonnefont J. Julien S. Marq-Lin N. Rodriguez I. Dubois-Dauphin M. (2010). Increased brain damage after ischaemic stroke in mice lacking the chemokine receptor CCR5. *Br. J. Pharmacol.* 160 311–321. 10.1111/j.1476-5381.2010.00697.x 20423342 PMC2874853

[B37] Stefan K. Kunesch E. Benecke R. Cohen L. G. Classen J. (2002). Mechanisms of enhancement of human motor cortex excitability induced by interventional paired associative stimulation. *J. Physiol.* 543 699–708. 12205201 10.1113/jphysiol.2002.023317 PMC2290505

[B38] Stefan K. Kunesch E. Cohen L. G. Benecke R. Classen J. (2000). Induction of plasticity in the human motor cortex by paired associative stimulation. *Brain* 123 572–584. 10686179 10.1093/brain/123.3.572

[B39] Stinear C. Petoe M. Byblow W. (2015). Primary motor cortex excitability during recovery after stroke: implications for neuromodulation. *Brain Stimul.* 8 1183–1190. 10.1016/j.brs.2015.06.015 26195321

[B40] Strube W. Bunse T. Malchow B. Hasan A. (2015). Efficacy and interindividual variability in motor-cortex plasticity following anodal tDCS and paired-associative stimulation. *Neural Plast.* 2015:10. 10.1155/2015/530423 PMC4381571 25866683

[B41] Suppa A. Biasiotta A. Belvisi D. Marsili L. La Cesa S. Truini A. (2013). Heat-evoked experimental pain induces long-term potentiation-like plasticity in human primary motor cortex. *Cereb. Cortex* 23 1942–1951. 10.1093/cercor/bhs182 22744704

[B42] Suppa A. Quartarone A. Siebner H. Chen R. Di Lazzaro V. Del Giudice P. (2017). The associative brain at work: evidence from paired associative stimulation studies in humans. *Clin. Neurophysiol.* 128 2140–2164. 10.1016/j.clinph.2017.08.003 28938144

[B43] Takahashi H. Xia P. Cui J. Talantova M. Bodhinathan K. Li W. (2015). Pharmacologically targeted NMDA receptor antagonism by nitromemantine for cerebrovascular disease. *Sci. Rep.* 5:14781. 10.1038/srep14781 26477507 PMC4609936

[B44] Tarri M. Brihmat N. Gasq D. Lepage B. Loubinoux I. De Boissezon X. (2018). Five-day course of paired associative stimulation fails to improve motor function in stroke patients. *Ann. Phys. Rehabil. Med.* 61 78–84. 10.1016/j.rehab.2017.11.002 29274471

[B45] Trotman M. Vermehren P. Gibson C. L. Fern R. (2015). The dichotomy of memantine treatment for ischemic stroke: dose-dependent protective and detrimental effects. *J Cereb. Blood Flow Metab.* 35 230–239. 10.1038/jcbfm.2014.188 25407270 PMC4426739

[B46] Volz M. S. Finke C. Harms L. Jurek B. Paul F. Flöel A. (2016). Altered paired associative stimulation-induced plasticity in NMDAR encephalitis. *Ann. Clin. Trans. Neurol.* 3 101–113. 10.1002/acn3.277 26900584 PMC4748309

[B47] Wiethoff S. Hamada M. Rothwell J. C. (2014). Variability in response to transcranial direct current stimulation of the motor cortex. *Brain Stimul.* 7 468–475. 24630848 10.1016/j.brs.2014.02.003

[B48] Witte A. V. Kürten J. Jansen S. Schirmacher A. Brand E. Sommer J. (2012). Interaction of BDNF and COMT polymorphisms on paired-associative stimulation-induced cortical plasticity. *J. Neurosci.* 32 4553–4561. 10.1523/JNEUROSCI.6010-11.2012 22457502 PMC6622078

[B49] Wolters A. Sandbrink F. Schlottmann A. Kunesch E. Stefan K. Cohen L. G. (2003). A temporally asymmetric hebbian rule governing plasticity in the human motor cortex. *J. Neurophysiol.* 89:2339. 12612033 10.1152/jn.00900.2002

[B50] Xia P. Chen H. S. V. Zhang D. Lipton S. A. (2010). Memantine preferentially blocks extrasynaptic over synaptic nmda receptor currents in hippocampal autapses. *J. Neurosci.* 30 11246–11250. 10.1523/JNEUROSCI.2488-10.2010 20720132 PMC2932667

[B51] Ziemann U. Iliać T. V. Pauli C. Meintzschel F. Ruge D. (2004). Learning modifies subsequent induction of long-term potentiation-like and long-term depression-like plasticity in human motor cortex. *J. Neurosci.* 24 1666–1672. 14973238 10.1523/JNEUROSCI.5016-03.2004 PMC6730462

[B52] Ziemann U. Lonnecker S. Steinhoff B. J. Paulus W. (1996). Effects of antiepileptic drugs on motor cortex excitability in humans: a transcranial magnetic stimulation study. *Ann. Neurol.* 3 367–368. 10.1002/ana.410400306 8797526

[B53] Ziemann U. Reis J. Schwenkreis P. Rosanova M. Strafella A. Badawy R. (2014). TMS and drugs revisited 2014. *Clin. Neurophysiol.* 126 1847–1868. 10.1016/j.clinph.2014.08.028 25534482

