# Supplementary material for: Paired Associative Stimulation as a Tool to Assess Plasticity Enhancers in Chronic Stroke
Source: Front Neurosci. 2019 Aug 2;13:792. doi: 10.3389/fnins.2019.00792 (PMC6687765; doi:10.3389/fnins.2019.00792)
Supplement: Supplementary file 1 [file Table_1.docx]

| **Supplementary Table 1. Post/Pre MEP Ratio*** | | | | | | |
| --- | --- | --- | --- | --- | --- | --- |
| Dose  (mg) | Responder Status | Mean ± Standard Deviation | Median | Minimum | Maximum | Inter Quartile Range |
| 0 | Non responders | 0.83 ± 0.15 | 0.83 | 0.612 | 1.04 | 0.15 |
| 0 | Responders | 2.71 ± 1.20 | 2.23 | 1.67 | 5.01 | 1.51 |
| 5 | Non responders | 3.46 ± 6.22 | 1.11 | 0.29 | 16.09 | 1.29 |
| 5 | Responders | 1.41 ± 0.86 | 1.17 | 0.59 | 2.92 | 1.16 |
| 10 | Non responders | 1.55 ± 0.73 | 1.67 | 0.54 | 2.34 | 1.08 |
| 10 | Responders | 1.20 ± 0.45 | 1.18 | 0.55 | 1.86 | 0.65 |
| 15 | Non responders | 1.49 ± 0.77 | 1.27 | 0.41 | 2.51 | 0.79 |
| 15 | Responders | 1.64 ± 0.69 | 1.59 | 0.63 | 2.74 | 0.98 |
| 20 | Non responders | 1.24 ± 0.71 | 1.29 | 0.34 | 2.00 | 1.16 |
| 20 | Responders | 1.31 ± 0.84 | 1.11 | 0.33 | 2.72 | 1.24 |

*Post PAS MEP amplitude averaged for 0-60 minutes.
